# Predicting Stroop Effect from Spontaneous Neuronal Activity: A Study of Regional Homogeneity

**DOI:** 10.1371/journal.pone.0124405

**Published:** 2015-05-04

**Authors:** Congcong Liu, Zhencai Chen, Ting Wang, Dandan Tang, Glenn Hitchman, Jiangzhou Sun, Xiaoyue Zhao, Lijun Wang, Antao Chen

**Affiliations:** 1 Key laboratory of Cognition and Personality of Ministry of Education, Faculty of Psychology, Southwest University, Chongqing, China; 2 Laboratory of Cognition and Mental Health, Chongqing University of Arts and Sciences, Chongqing, China; University of Texas at Dallas, UNITED STATES

## Abstract

The Stroop effect is one of the most robust and well-studied phenomena in cognitive psychology and cognitive neuroscience. However, little is known about the relationship between intrinsic brain activity and the individual differences of this effect. In the present study, we explored this issue by examining whether resting-state functional magnetic resonance imaging (rs-fMRI) signals could predict individual differences in the Stroop effect of healthy individuals. A partial correlation analysis was calculated to examine the relationship between regional homogeneity (ReHo) and Stroop effect size, while controlling for age, sex, and framewise displacement (FD). The results showed positive correlations in the left inferior frontal gyrus (LIFG), the left insula, the ventral anterior cingulate cortex (vACC), and the medial frontal gyrus (MFG), and negative correlation in the left precentral gyrus (LPG). These results indicate the possible influences of the LIFG, the left insula, and the LPG on the efficiency of cognitive control, and demonstrate that the key nodes of default mode network (DMN) may be important in goal-directed behavior and/or mental effort during cognitive control tasks.

## Introduction

The ability to focus resources on goal-relevant information while filtering out or inhibiting irrelevant information is crucial for academic and career successes. However, there are large individual differences in this ability. For example, some people can control their game impulsion when they are working, whereas others cannot suppress their craving, despite being aware of the danger of losing their jobs. From the viewpoint of cognitive psychology, such variation refers to the inter-individual differences in cognitive control, and a massive amount of evidence suggests that this function is attributed to the purview of the frontal lobes [1–4]. One elegant probe of the integrity and neural underpinnings of cognitive control is provided by the conflict effect on interference tasks, such as the Stroop task [5].

In a typical Stroop task, participants are required to name the font color of a given word that spells a color name. The responses to incongruent stimuli (I, e.g., the word RED printed in blue) are slower and less accurate in comparison to congruent stimuli (C, e.g., the word RED printed in red). This decline in performance is termed the Stroop effect, which is thought to reflect the cost of recruitment of cognitive control resources necessary for resolving the interference from conflicting stimulus information [6]. This effect has been applied in clinical neuropsychology to explore specific cognitive and neural dysfunction in psychiatric patients [4,7]. For example, individual differences in the Stroop effect have been demonstrated to predict future development of Alzheimer’s disease (AD) [8]. For the researchers in the basic cognitive neuroscience, it offers the opportunity to study interference and attention control [6,7,9–11]. Pursuing this approach, numerous neuroimaging studies have investigated the neural basis of the conflict resolution process in Stroop tasks and have found that cognitive control is linked to multiple brain regions associated with attention, response inhibition and motor control [12–16]. The most consistent activations have been observed in the dorsal anterior cortex (dACC), the inferior frontal gyrus (IFG) and the response organization regions, including the supplementary motor areas (SMA) and the pre-supplementary motor areas [12–16]. It has been suggested that the LIFG appeared to be associated with the function of selection of semantic knowledge among competing alternatives via biasing or gating relevant information for posterior areas [17–19]. Most existing studies indicate that the dACC is involved in conflict monitoring in the Stroop task [1,20–22]. The SMA and pre-SMA are associated with the selection and execution of responses [23, 24]

Though informative, the previous studies often ignored the importance of variability across individuals. However, the data of individual differences may provide novel insights into the neural substrates of the Stroop effect [25, 26]. In addition, when dealing with abnormal neural and psychological processes in clinical settings, an understanding of such individual differences can offer more information than the commonalities in function across individuals [27]. Recently, some researchers have suggested that it is important for understanding brain function to explore intrinsic brain activity which consumes more than 90% of the brain’s energy [28,29]. A huge number of positives have been attributed to functional activation paradigms. However, from the brain energy metabolism perspective, the task-evoked activity may reveal only a small fraction of the actual functional activity performed by our brain [28]. In addition, a sole focus on task-evoked activity may ignore the alternative possibility that brain’s operations are mainly intrinsic [28] and existing studies have shown that individual variability in behaviors can be predicted from intrinsic activity [30–34]. For example, Wang et al. (2014) found that intrinsic activity could predict subjects’ conflict adaption performance in a Flanker task. Furthermore, as there is no task during image acquisition, studies may avoid concerns about differences in experiment design and task strategy [35].

To date, only one study has examined intrinsic neural basis of individual variability in the Stroop effect [31]. Nevertheless, this study used region of interest (ROI) analysis and the analyses were limited a set of 31 predefined ROIs. Therefore, they may ignore some critical brain areas. In addition, they took the average correlation within the network as the index of the status of the network. Thus, it is sometimes difficult to reveal the regions whose properties within the network are especially important to the performance of a particular cognitive task. Therefore, the present study attempted to explore the intrinsic functional underpinnings of individual differences in the Stroop effect at the whole brain level.

It has been suggested that the analysis of rs-fMRI signals is useful for elucidating the “intrinsic” functional architectures of human brain [28,35,36]. Regional homogeneity (ReHo) analysis is a profitable method for investigating regional properties of the intrinsic brain dynamics at the whole brain level [37]. It indexes the similarities between the time series of a given voxel and its nearest neighbors. Previous investigations have indicated that the ReHo has biological relevance: the ReHo of large portion of the grey matter in the brain is very stable across time and can naturally reflect the functional organization of the cortex [38], and individuals with cognitive brain disorders show abnormal ReHo in the regions important to corresponding cognitive processes [39–42]. Furthermore, several recent studies have demonstrated that ReHo-behavior correlation analysis can be useful to explore the neural basis of individual variations in behavior [30,32–34]. For instance, Wang et al. [34] observed significant correlations between the conflict adaption effect and the ReHo values in the left dorsolateral prefrontal cortex. Thus, it appears that the regional properties of intrinsic brain dynamics can reliably reflect aspects of cognitive function.

In the present study, we attempted to investigate the special brain regions which could predict individual differences in the Stroop effect using rs-fMRI. For the regional properties, brain ReHo was calculated during resting state, which indexes the local synchronization within a brain region [37]. We performed a correlation analysis between participants’ performance in the Stroop task and their ReHo values to uncover potential core regions that could account for individual variations in the Stroop effect. Researchers have suggested that ReHo variations can reflect individual differences in cognition and behavior [43]. Moreover, it has been suggested that resting state brain activity reflects task-evoked activity of brain network [44]. Therefore, we predicted that the ReHo values of voxels in the regions subserving conflict resolution (e.g., the dACC, IFG, SMA and/or pre-SMA) may be significantly correlated with the Stroop effect.

## Materials and Methods

### Ethics Statement

Approval of the study was made by the Human Research Ethics Committee of the Southwest University of China, and all participants provided written informed consent

### Subjects

Forty-four healthy, right-handed college students (34 females; mean age = 18.9 years, *SD* = 0.8) from Southwest University, China, were recruited for this study as paid participants. All of them were Chinese native speakers and naive to the purposes of the experiment. All subjects had normal or corrected-to-normal vision, without achromatopsia or color weakness. Firstly, each subject underwent a brief resting- state scan during which they were required to relax with their eyes closed. Each subject then performed a Stroop task outside the scanner. The data of three subjects was excluded due to excessive head movement artifacts (which exceeded 2 mm in transition or 2 degrees in rotation).

### Apparatus and Procedure

The experiment was carried out on a PC connected to a VGA color monitor, operating at a frame rate of 85 Hz with a spatial resolution of 1,024 × 768 pixels. The stimuli were presented using E-Prime Software (Psychology Software Tools, Inc. Pittsburgh, PA). RTs and error rates were recorded by computer. The viewing distance was approximately 60 cm. Stimuli were the standard Stroop color words, consisting of four Chinese characters “Hong” (red), “Huang” (yellow), “Lan” (blue) and “Lv” (green). Each character was presented in one of the four colors (i.e., red (255, 0, 0), yellow (255, 255, 0), green (0, 255, 0), blue (0, 0, 255); 16 stimuli altogether). The display background was always black.

Each trial started with a white fixation in the center of the screen for 300 ms. Next, the character printed in color was displayed for 1400 ms or until a response was made, which was followed by a black interval for 200~400 ms randomly. Then the next trial started. Subjects were instructed to respond according to the printed color of the character by pressing the ‘D’ key with their left middle finger if the color word was printed in red, the ‘F’ key with the left index finger if the color word was printed in green, the ‘J’ key with the right index finger if the color word was printed in yellow, and the ‘K’ key with the right middle finger if the color word was printed in blue. A familiarization session was conducted to allow subjects to adapt the task, which consisted of 24 trials of the same type as in the main experiment. In the main experiment, 240 trials were presented in 2 blocks of 120 trials, with a mandatory 30-second break for rest between two blocks. Each block consisted of 60 congruent trials and 60 incongruent trials, which were presented randomly.

### Behavioral data analysis

Firstly, we calculated mean RTs and accuracies for each condition(C, I); the calculation of mean RTs excluded data from error trials and outlier trials (more than 2.5 standard deviations [*SD*s] from the mean, calculated for each condition separately). Then, the mean RT (the average of I and C separately) and the Stroop effect (I minus C) were computed for each subject. Finally, the ratio of the Stroop effect over mean RT was computed to control for general response latency differences, and we named this ratio as the Stroop effect _corrected_. The Stroop effect _corrected_ was thought to be capable of providing a more suitable behavioral index than the original Stroop effect when a brain-behavior correlation analysis was conducted [45].

### Image acquisition and analysis

Images were acquired with a Siemens (3.0 Tesla) scanner. An Echo-Planar imaging (EPI) sequence was used for data collection, and 240 T2-weighted images were recorded per run (TR = 2000 ms; TE = 30 ms; flip angle = 90°; FoV = 200 × 200 mm^2^; matrix size = 64 × 64; 33 interleaved 3 mm-thick slices; in-plane resolution = 3.13 × 3.13 mm^2^; interslice skip = 0.6 mm). T1-weighted images were recorded with a total of 128 slices at a thickness of 1.33 mm (TR = 2530 ms; TE = 3.39 ms; flip angle = 7°; FoV = 256 × 256 mm^2^). During the resting state, subjects were told to keep awake with their eyes open and as motionless as possible and not concentrate on anything in particular.

### Data preprocessing

SPM8 (Wellcome Department of Cognitive Neurology, London, UK, http://www.fil.ion.ucl.ac.uk/spm/spm8) was used to pre-process the functional images [46]. The first 5 images were discarded to ameliorate the magnetization equilibrium and adapt subjects to the environment. The remaining functional images were corrected for interleaved acquisition, and then realigned to estimate and modify the six parameters for head movement. A mean functional image was then obtained for each subject. To normalize functional images, each subject’s T1-weighted images were co-registered to the mean functional images and were subsequently segmented into gray matter (GM), white matter (WM) and cerebrospinal fluid (CSF). The parameters obtained in segmentation were used to normalize each participant’s functional images onto the Montreal Neurological Institute space in 3 × 3 × 3 mm^3^ resolution.

To further exclude the residual effect of motion on the relationship between ReHo and Stroop effect _corrected_, the mean framewise displacement (FD) proposed by Jenkinson was firstly computed for 41 subjects separately. Subjects with excessive motion (more than 3 *SDs* from the mean FD; mean FD: 0.056 ± 0.021) were excluded as outliers. No subjects were excluded due to extreme FD.

### ReHo analysis

Before ReHo calculation, through linear regression, the influences of linear trends were removed from the normalized EPI images and then the low frequency drift and high-frequency noise were also filtered out by a band-pass filter (0.01–0.08 Hz) [36,47]. In addition, nuisance correction was conducted by regressing out 6 motion signals as well as whiter matter and cerebrospinal fluid signals. Then, following Zang et al. [37], ReHo was performed on a voxel-by-voxel basis by calculating Kendall’s coefficient of concordance [48] of the time series of a given cluster of neighboring voxels. Here, cubic clusters of 27 voxels (corner connection) were used and the ReHo value of every cubic cluster was assigned to the central voxel [37]. The ReHo images were then smoothed with a full width at half maximum (FWHM) of 6 × 6 × 6 mm^3^. The larger ReHo value for a given voxel, the higher local synchronization of rs-fMRI signals among neighboring voxels was. All of these procedures were performed using the Resting-state fMRI date analysis Toolkit (REST) and Data Processing Assistant for Resting-State fMRI (DPARSF) software [37,49] (http://www.restfmri.net/forum/).

### ReHo-Stroop effect correlation analysis

A partial correlation analysis was carried out in a voxel-wise manner to examine the relationship between ReHo values and the Stroop effect _corrected_, while controlling for age, sex and FD. In order to correct for multiple comparisons, Monte Carlo simulations were performed. The parameters were as follows: individual voxel threshold probability = 0.01, 1,000 simulations, 2 sided, FWHM estimated by 6 mm FWHM, cluster connection radius = 5 mm (edge connected), with a grey matter mask. These procedures were performed using the AlphaSim program in the REST software. The AlphaSim correction was achieved by setting the cluster size and individual voxel height threshold. For example, according to the simulations, the activation clusters showing a corrected significant correlation (*p* < 0.05) between ReHo and the Stroop effect _corrected_ were extracted from statistic images with cluster size > 34 voxels and a voxelwise *p* value of < 0.01.

## Results

### Behavioral performance

The discarded trials occupied 9.63% of the all trials (error trials: 7.32% and outlier trials: 2.31%). In order to testing whether there existed speed-accuracy trade-off, we performed a correlation analysis between general RT and general accuracy rates. If there existed speed-accuracy trade-off,subjects would produce faster response but make more errors. That is to say, if there existed speed-accuracy trade-off, a significant positive correlation between general RT and general accuracy rates would be observed. The correlation analysis revealed a significant negative correlation (*r* = -0.372, *p* < 0.05) between general RT and general accuracy rates. This meant that there was minimal trade-off between speed and accuracy rates.

**Fig 1:**
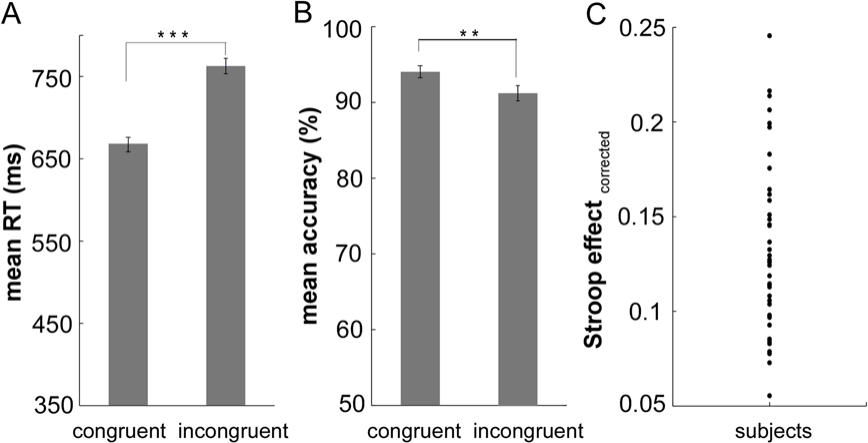
The behavioral results. Pane lA illustrates the mean RT as a function of congruency (C, I). It indicates a significant Stroop effect (I-C), with some subjects exhibiting a larger Stroop effect than others. Pane lB illustrates that the mean accuracy rate as a function of congruency (C, I). Error bars represent standard errors, respectively. N = 41. RT = response time. (*** *P* < 0.001; * * *P* < 0.01). Pane lC represents individual differences related to Stroop effect (ratio of Stroop effect over mean RT). Note: each circle represents a subject’s Stroop effect score.

The mean RTs and accuracies are shown in Fig 1. A paired-samples *t*-test revealed that responses were significantly faster in the congruent condition (668ms, *SD* = 49 ms) than in the incongruent condition (763 ms, *SD* = 60 ms; *t*(40) = -17.9, *p* < 0.001; see Fig 1A). The accuracies were higher in the congruent condition (94.06%, *SD* = 3.69%) than in the incongruent condition (91.30%, *SD* = 5.84%; *t*(40) = 3.44, *p* < 0.01; see Fig 1B). Thus, the results indicated a significant Stroop effect. However, as shown in Fig 1C, there was a large amount of individual variability in this effect.

### ReHo-Stroop effect correlation analysis

**Fig 2:**
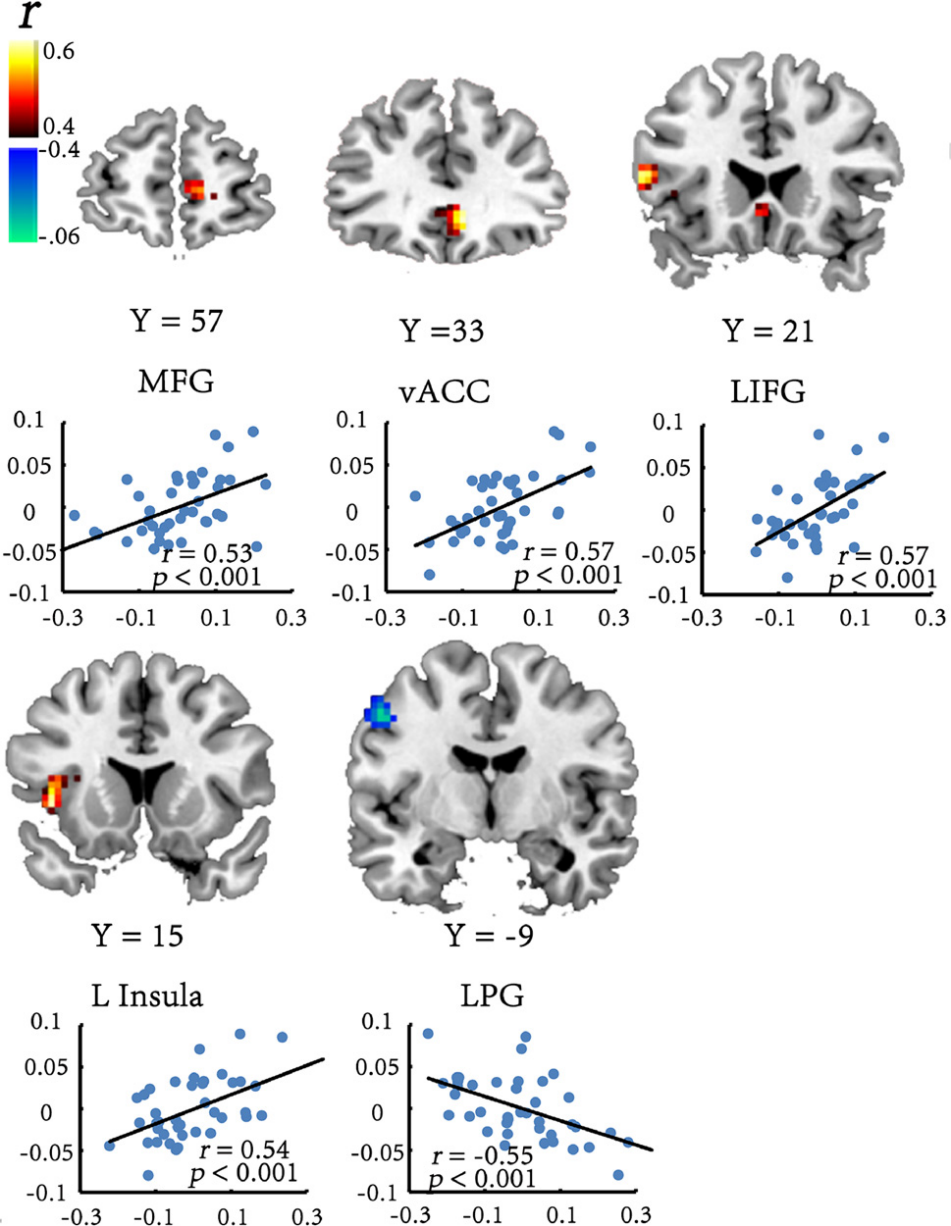
Brain regions which exhibit significant correlations between ReHo and participants’ cognitive control efficiency [expressed as (I-C)/mean RT] in the Stroop task. The numbers at the bottom of each image refer to the y-coordinates of the Montreal Neurological institute (MNI). The threshold was set at *p* < 0.05 (corrected). Each scatter plot shows the correlation between the cognitive control efficiency and averaged ReHo in the corresponding region with gender, age and FD controlled. x-axis, ReHo value; y-axis, Stroop effect. Each dot represents data from one participant.

At a threshold of *p* < 0.05 (corrected), significant positive correlations between ReHo and the Stroop effect _corrected_ were observed in the left IFG, the left insula, the ventral anterior cingulate cortex (vACC) and medial frontal gyrus (MFG). Significant negative ReHo-Stroop effect _corrected_ correlations were observed in the left precentral gyrus (LPG) (Table 1 and Fig 2). We conducted a partial correlation analysis between accuracy rates and ReHo values of the mentioned brain regions above while controlling gender, age and FD. None significant correlation between accuracy rates and ReHo values was found in the mentioned areas (for more information, see S1 Text). There may be a celling effect in the accuracy rates, which made it difficult to find any correlation in the present study.

**Table 1:** Brain regions which exhibit significant correlations between ReHo and Stroop effect.

Region	BA	Cluster size	MNI coordinate (peak:xyz)			*r*(peak)
vACC	24/32	1863	9	33	-6	0.59
MFG	10/32	945	12	57	3	0.52
L insual	13	1134	-42	15	0	0.59
L PG	4/6	972	-51	-9	45	-0.59
L IFG	45	918	-57	21	12	0.55

Note: The threshold was *p* < 0.05 (corrected). IFG = Inferior Frontal Gyrus; PG = Precentral Gyrus; vACC = Ventral Anterior Cingulate Cortex; MFG = Medial Frontal Gyrus; L = left; ReHo = Regional Homogeneity; Stroop effect [RT _(incongruent minus congruent)/mean RT_, ms].

Recent studies have suggested that the task conflict and information conflict exist in the Stroop task [10]. Task conflict was defined as the latency difference between color words and non-letter neutrals. Informational conflict was defined as the latency difference between incongruent and congruent trials. Furthermore, these two types of conflicts are processed differently [50,51]. In order to examine whether only one of these processes is correlated with the regions found in the ReHo-behavior correlation analysis, a partial correlation analysis was conducted between the Stroop congruent and Stroop incongruent (separately) RT and ReHo values of the mentioned brain regions above while controlling gender, age and FD. None significant correlation was found in the mentioned areas (For more information, see S2 Text).

## Discussion

The present study adopted the ReHo approach to investigate the intrinsic functional underpinnings of individual differences in the Stroop effect _corrected_ at the whole brain level. Behavioral results showed that there were individual differences in the Stroop effect _corrected_. Further, the individual differences could be predicted by functional homogeneity within the local region. Unsurprisingly, the ReHo values of voxels in the regions subserving conflict resolution (the LIFG, the left insula, and the LPG) were significantly correlated with the Stroop effect _corrected_. Less expectedly, the significant correlations were observed in the regions (vACC and MFG) exhibiting task-induced deactivations during goal-directed behaviors. To the best of our knowledge, this is the first study to investigate the association between the individual differences in the whole-brain intrinsic functional architecture and the Stroop effect _corrected_.

The ReHo in the LIFG was significantly correlated with the Stroop effect _corrected_, which was consistent with the results from task-based fMRI studies. For instance, the activation in the LIFG has been reported as being greater in the incongruent trials than in the congruent trials [52], indicatingj that the LIFG was involved in the conflict resolution. In addition, in the lesion study by Hamilton and Martin [53], the patients with circumscribed LIFG lesions exhibited especially higher error rates in the incongruent condition compared to healthy individuals and other frontal lobe lesion patients whose LIFGs were undamaged. These studies consistently suggest that the LIFG is critical for cognitive control. Regarding the specific role of the LIFG in the Stroop effect, it has been suggested that the LIFG facilitates the selection of semantic knowledge among competing alternatives based on task demands via biasing or gating relevant information from posterior areas (e.g., temporal lobe, occipital lobe) [17–19]. Together, it seems that the LIFG contributes to resolving the Stroop interference effect through influencing the semantic processing. However, other researchers have speculated that the LIFG may play an important role in response inhibition via its indirect connection to the motor system in the response selection and execution phase [54]. Further elucidation of the role of the LIFG will require exploring the functional circuits associated with the LIFG by calculating the intrinsic functional connectivity of LIFG.

The ReHo values in the left insula also had a significant correlation with the Stroop effect size. This result suggests that the left insula plays an important role in facilitating conflict resolution. A related study by Leung et al. [55] showed that the left insula was more active in the incongruent trials than in the congruent trials, indicating that the Stroop interference was linked to increased activity in the insula. In another study, it has been found that the left insula was activated in the Go/no go and stop-signal tasks, which require subjects to inhibit the prepotent but inappropriate responses [56,57]. Furthermore, a structural MRI study found that the insula thickness was positively correlated with impulsivity and impaired planning capacity [58]. Overall, the evidence suggests that ReHo-behavior correlation in the left insula may reflect the individual differences in inhibiting responses to the task-irrelevant information.

The correlation between the ReHo values in the PG and the Stroop effect _corrected_ may be due to its important role in the motor system. It is part of the primary motor cortex that is a natural focus for the investigation of changes associated with motor skill acquisition [59]. Furthermore, it has consistently been observed to be interconnected with the SMA [60–63], which have been considered to be involved in response selection and the execution of responses [23,24], indicating the important position of the PG in the motor control circuits. A neuroimaging study found that along with a reduction in behavioral RT, the activity within the motor cortex including the PG increased after a 4–2 mapping practice-related Stroop task [64]. Thus, the involvement of the PG during the Stroop task may be related to motor skill learning of task-relevant S–R mapping, which would affect the efficiency of response execution.

Less expectedly, ReHo values in the vACC and the MFG were significantly positively correlated with the Stroop effect _corrected_. The previous task-based studies found that the vACC is involved in conflict resolution on some tasks [65–67]. Nevertheless, these studies always employed the emotional interference task. Recently, some studies have showed that the vACC and MFG are key nodes of the DMN [68–71], which has been associated with spontaneous cognition [72]. In terms of cognitive control, the DMN facilitates the processing of internal mental noise, which is an interference source in attention-demanding tasks [73]. Therefore, the significant positive correlations between the Stroop effect and the ReHo values in the vACC and the MFG may mean that the individuals with higher synchronization of spontaneous activity in the vACC and the MFG will suffer stronger interference from internal noise.

Intriguingly, there was no significant correlation between ReHo and the Stroop effect _corrected_ in the dACC that is most frequently associated with cognitive control [10]. Certain methodological factors may have contributed to these negative results. First, the differences of experimental materials may offer an explanation. In our study, the lexical control conditions were employed. However, the most of studies that have found dACC activation in the Stroop task have employed nonverbal conditions (e.g. Colored crosses or colored blocks), which have different stimulus attributes and processing requirements, when compared with a verbal interference condition [13,74–77]. But lexical control conditions often do not produce increased dACC activation [16]. Second, the proportion of congruence may affect the results. Some researchers found that the dACC was selectively activated only on the incongruent trials during high expectancy congruent blocks [12]. This result was attributed to the putative role of the dACC in anticipating response conflict or brokering strategy shifts. However, in our design format, the proportion of the two experimental conditions was equal, thereby minimizing the effects of expectancies. Additional studies are needed to elucidate the role of the dACC.

Taken together, the ReHo index-Stroop effect correlation analysis suggests that the cognitive control is linked to multiple brain regions associated with attention, response inhibition and motor control. These findings are consistent with previous task-based fMRI studies [12–16] but also extend imaging studies on cognitive control in important ways. Most of previous task-based fMRI studies focus on the regions that are more active in the incongruent trials than in the congruent trials, but our findings suggest that individuals’ behavioral performance in cognitive tasks may be affected by the regions exhibiting task-induced deactivations during goal-directed behaviors.

Notwithstanding its implications, there were several limitations in this study. First, the Stroop effect is rather complicated, which has been identified to consist of multiple components, such as task conflict and information conflict (semantic and response conflicts) [50]. Therefore, although the present results provide some insights for the individual differences of this effect, the corresponding interpretations are not exclusive. In the future work, more attention should be paid on the spontaneous brain activtiy associated with these subcomponents of the Stroop effect. Second, this study only focused on college-age group. The inclusion of other age groups in the further work would help to clarify whether findings generalize to other groups. Finally, numerous studies have found that the cognitive control were associated with two relatively separate networks of multiple brain regions: the adaptive (frontoparietal) and stable (cingulo-opercular) network [78–80] and these two networks obviously have good overlap with regions subserving conflict resolution. But the long-distance interregional connectivity patterns within and across these two networks were not assessed and should be addressed in future work.

## Conclusion

In summary, we employed the ReHo method to investigate the intrinsic functional underpinnings of individual differences in Stroop effect at the whole brain level. Significant correlations were observed between Stroop effect size and ReHo values in the LIFG, the left insula, the vACC, the MFG and the LPG. The present findings indicate that: 1) multiple brain regions are involved in the Stroop task; 2) the ReHo index of rs-fMRI signals could be used to predict individuals’ cognitive task performance; 3) the key nodes of DMN may be important in goal-directed behavior and/or mental effort during cognitive tasks. Finally, our findings have implications in clinical settings by examining the brain’s intrinsic functional architecture in the identification of biomarkers for AD, and in the assessment of AD.

## Supporting Information

S1 Text Correlation analysis between ReHo and accuracy rates. (DOCX)

S2 Text Correlation analysis between ReHo and the RT in the incongruent and congruent trials (separately). (DOCX)
