# Supplementary material for: Predicting Stroop Effect from Spontaneous Neuronal Activity: A Study of Regional Homogeneity
Source: PLoS One. 2015 May 4;10(5):e0124405. doi: 10.1371/journal.pone.0124405 (PMC4418763; doi:10.1371/journal.pone.0124405)
Supplement: S1 Text — (DOCX) [file pone.0124405.s001.docx]

**S1 Text Correlation analysis between ReHo and accuracy rates**

Firstly, we identified regions of interest (ROIs) based on the regional ReHo-behavior correlation results and extracted ReHo values from the ROIs of each subjects. Then, the partial correlation analysis was performed between the ReHo values of each ROI and the accuracy rates separately while controlling gender, age and framewise displacement. We found none significant correlation between accuracy rates and ReHo values in the mentioned areas (vACC: *r* = 0.302, *p* = 0.066; MFG: *r* = 0.245, *p* = 0.138; IFG: *r* = -0.100, *p* = 0.552; Insula: *r* = 0.115, *p* = 0.494; PG: *r* = -0.083, *p* = 0.622).
