# Supplementary material for: Predicting Stroop Effect from Spontaneous Neuronal Activity: A Study of Regional Homogeneity
Source: PLoS One. 2015 May 4;10(5):e0124405. doi: 10.1371/journal.pone.0124405 (PMC4418763; doi:10.1371/journal.pone.0124405)
Supplement: S2 Text — (DOCX) [file pone.0124405.s002.docx]

**S2 Text Correlation analysis between ReHo and the RT in the incongruent and congruent trials (separately)**

Firstly, we identified regions of interest (ROIs) based on the regional ReHo-behavior correlation results and extracted ReHo values from the ROIs of each subjects. Then, the partial correlation analysis was performed between the ReHo values of each ROI and the Stroop congruent and incongruent (separately) RT while controlling gender, age and framewise displacement. We found none significant correlation between RTs and ReHo values in the mentioned areas (Stroop congruent RT: vACC: *r* = -0.076, *p* = 0.649; MFG: *r* = -0.009, *p* = 0.958; IFG: *r* = -0.025, *p* = 0.881; Insula: *r* = -0.189, *p* = 0.256; PG: *r* = 0.025, *p* = 0.880. Stroop incongruent RT: vACC: *r* = 0.204, *p* = 0.219; MFG: *r* = 0.256, *p* = 0.121; IFG: *r* = 0.249, *p* = 0.131; Insula: *r* = 0.084, *p* = 0.615; PG: *r* = -0.241, *p* = 0.145).

The results may imply that the overall RT in the incongruent and congruent trials cannot index the conflict control recruited by Stroop effect _corrected_. The overall RT in the congruent and incongruent trials may comprise multiple cognitive componential. For example, in the incongruent trials, the task conflict and information conflict add up in a sub-additive way. Furthermore, the previous studies have pointed out that the task conflict and information conflict are probably mediated by multiple, independent conflict control loops (Braverman & Meiran, 2010; Goldfarb & Henik, 2007; Kalanthroff et al., 2012; Steinhauser & Huebner, 2009). Therefore, the overall incongruent RT is not a good indicator for the conflict control loop recruited by Stroop effect _corrected_.
